# Screening Donated Blood for Transfusion Transmitted Infections by Serology along with NAT and Response Rate to Notification of Reactive Results: An Indian Experience

**DOI:** 10.1155/2014/412105

**Published:** 2014-11-16

**Authors:** Rahul Chaurasia, Shamsuz Zaman, Bankim Das, Kabita Chatterjee

**Affiliations:** Department of Transfusion Medicine, All India Institute of Medical Sciences, New Delhi 110029, India

## Abstract

*Background*. Transfusion safety begins with healthy donors. A fundamental part of preventing transfusion transmitted infections (TTIs) is to notify and counsel reactive donors. Donor notification and counselling protect the health of the donor and prevent secondary transmission of infectious diseases. *Methods*. 113,014 donations were screened for TTIs, namely, HIV, HBV, HCV, and syphilis, by serology and nucleic acid testing. All reactive donors were retested (wherever possible) and notified of their status by telephone or letter. All initial reactive screens were followed over six months. *Results*. We evaluated 2,838 (2.51%) cases with reactive screening test results (1.38% HBV, 0.54% HCV, 0.27% HIV, and 0.32% syphilis). Only 23.3% of donors (662) responded to notification. The response among voluntary donors was better as compared to the replacement donors (43.6% versus 21.2%). Only 373 (56.3%) responsive donors followed their first attendance at referral specialties. Over six months, only 176 of 662 (26.6%) reactive donors received treatment. *Conclusion*. Our study shed light on the importance of proper donor counselling and notification of TTI status to all reactive donors who opt to receive this information. There is also an urgent need to formulate the nationally acceptable guidelines for notification and follow-up of reactive donors.

## 1. Introduction

Blood transfusion is safer than ever before through continuous improvements in donor recruitment, screening, testing of donated blood with increasingly sensitive assays, and appropriate clinical use of blood [1]. Serologic testing for transfusion transmitted diseases had historically been the foundation of blood screening, while newer strategies like nucleic acid testing (NAT) have helped further shorten the “window period” [2]. Currently, no technology exists to completely detect all window period donations. No matter how sensitive NAT becomes, we will never be able to completely close the exposure-to-seroconversion window period. The general public and media might believe that with the advancement in testing technologies zero risk blood products are currently available. This generalization is far from reality as judged by our current experience with new testing methodologies. Breakthrough transmissions of viruses (HIV-1 and HCV) had occurred as late as 2009 due to NAT failures because of low level of viraemia and/or suboptimal amplification efficiency [3]. Moreover, threat of infectious agents entering the blood supply is not static and may evolve as new pathogens emerge or as old ones change their epidemiological pattern [4]. Therefore, regardless of testing modality chosen, a nonzero risk of disease transmission still exists in all its seriousness [5]. Under current practice in India, potential blood donors, after registration, filling of donor-health questionnaire, and brief medical examination, are sent for predonation counseling. During counseling process of blood donation, postdonation care and the outcomes of donation are explained. After blood donation, samples are collected for screening for anti-HIV-1/2, anti-HCV, and HBsAg, RPR for syphilis, and slide/card test for malaria. In addition to ELISA, NAT is being increasingly used in many centers to further improve blood safety although it is not mandated by national authorities.

In 2002, the Government of India adopted the National Blood Policy* “An action plan for blood safety”* to ensure safe blood supply. This policy advocates notification to all reactive blood donors. Blood banks are thus now required to obtain written consent from donors at time of donation for screening blood for TTI (Transfusion-transmitted Infections) and whether they wish to be informed about their abnormal tests results [6]. If any of the screening tests are abnormal, before notification to the donors the tests are repeated either using two assays of differing principles or in duplicate with the same assay so as to avoid notification of false-positive results. Donors who report back to the transfusion facility are retested and if found repeat reactive are referred to integrated counseling and testing centers (ICTC) for HIV and gastroenterology and STD clinic for HBV/HCV and syphilis, respectively, for counseling, confirmatory testing, and management.

Currently in India, most of the blood banks including ours do not have the facility to perform confirmatory tests for TTI. The donors are informed only on the basis of their screening tests available in blood bank. As most of the donors do not expect to hear that they have reactive results they may become extremely distressed to hear this news. These donors may be highly motivated to donate, having desire to help others, or simply want some time off work or may have other motives. This, unfortunately, may leave the donor with a negative feeling towards blood donation or diminish his/her own self-worth [7, 8]. On the other hand, a small minority of individuals appear to ignore notification and continue to donate blood elsewhere. Some of the donors even use blood donations as a means for free testing because of their high risk behavior* (test seekers)* [9].

Donor notification can therefore be a challenging task demanding special skills from the staff involved who should always be prepared to meet new challenges and help donors come to terms with their newly discovered status. Although the blood policy advocates disclosure of TTI status, donors are not, in practice, informed about their results. The onus lies with the donor to contact the blood bank [10]. There is very little information available about donor behavior on receipt of reactive TTI results. We undertook this study to determine the response rate following notification of reactive status to the donors. We also assessed the prevalence of TTI using serology as well as NAT among blood donors attending our center.

## 2. Material and Methods

Between January 2011 and December 2013, 113,014 donations at Main Blood Bank, All India Institute of Medical Sciences, New Delhi, were screened by ELISA using 4th generation ELISA test kits for HIV-1/2, 3rd generation ELISA test for HBsAg and HCV infections, and rapid immunochromatographic test for syphilis. All donations were also screened individually using the Procleix Ultrio assay (Novartis, Emeryville, CA), a multiplex NAT assay for the detection of hepatitis B virus (HBV) DNA, hepatitis C virus (HCV) RNA, and human immunodeficiency virus-1 (HIV-1) RNA. If initial NAT result was positive, sample was retested again in triplicate. If any of these three tests were positive, sample was drawn from plasma bag and tested in triplicate. Discriminatory NAT was run if any test from plasma bag came positive. The analytical sensitivities of the Procleix Ultrio assay for HBV DNA, HCV RNA, and HIV-1 RNA are 10.4 (9.2–12.2) IU/mL, 3.0 (2.7–3.4) IU/mL, and 47.9 (43.3–54.5) IU/mL, respectively. The analytical sensitivities for the Procleix dHBV (d = discriminatory), dHCV, and dHIV assays are 8.5 (7.6–9.8) IU/mL, 3.2 (2.8–3.6) IU/mL, and 53.6 (47.9–61.2) IU/mL, respectively.

**Figure 1:**
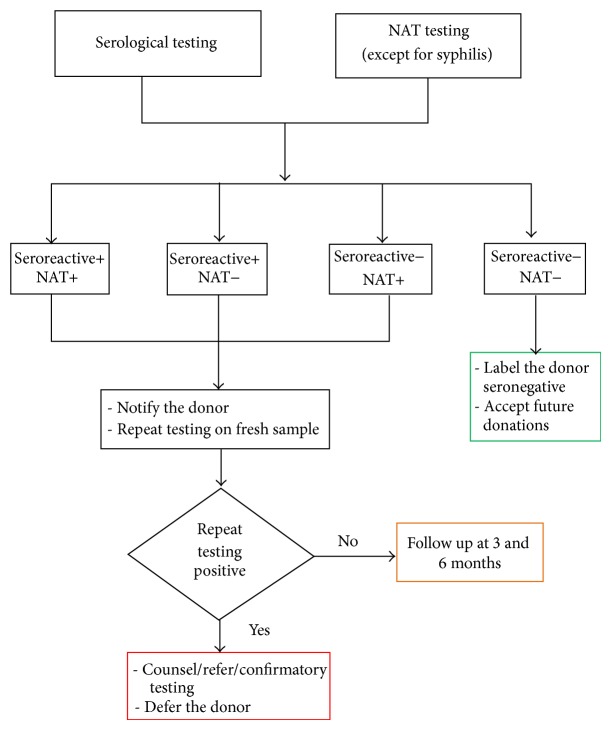
Algorithm for donor testing and recall.

If the results of either serology and/or NAT were found to be positive, blood unit was discarded as per hospital SOPs and donor was notified of his/her status either by telephone or by letter. The first follow-up call was made on the 10th day of notification. If the donor did not respond to this first call, second and third follow-up calls were made every 10 days. The case was closed only if the donor did not respond to any of the three telephone calls/letters and the case was labeled as nonresponder. Donors who responded to the call/letters and came back to transfusion facility were counseled and retested by ELISA with fresh blood sample. Donors whose results from fresh sample were concordant with earlier tests were referred to concerned clinical specialty and donors who tested nonreactive were asked to remain in follow-up (Figure 1).

## 3. Results

A total of 113,014 donors were evaluated comprising 85.4% replacement and 14.6% voluntary donors. The majority of the donors (97%) donated blood for the first time. The demographic details of donors are given in Table 1. Total of 2838 (2.51%) donors tested reactive for TTI. Prevalence of TTI was 1.38% for HBV, 0.54% for HCV, 0.27% for HIV, and 0.32% for syphilis (Table 2). For HIV, HBV, and HCV testing, concordant serological and NAT reactive results were found in 1643/2480 (66.25%) positive donations. NAT yield in our study was 1 in 628 donations (180 NAT+ELISA − cases) (Table 2). Of all the donors who were notified of their reactive status only 662 (23.3%) donors reported back to transfusion facility. The response among voluntary donors was better as compared to the replacement donors (43.6% versus 21.2%) (Table 3). Donors residing in the urban nearby areas responded better than those who lived in rural or far-off areas. Donor notification using telephone was more beneficial as more donors turned up to transfusion facility. Only 373 (56.3%) responsive donors followed their first attendance at referral specialties. Over six months, 176 of 662 (26.6%) responsive donors were undergoing treatment.

## 4. Discussion

With over 93 million donations made every year worldwide, blood transfusion continues to save millions of lives each year and improve the life expectancy and quality of life of patients suffering from life-threatening conditions [11]. At the same time, blood transfusion is an important mode of transmission of infection to the recipients. Prevalence of TTI in India is 1.8–4%, 0.4–1.09%, 0.2–1%, and 0.05–0.9% for HBV, HCV, HIV, and syphilis, respectively [12–17]. Prevalence of TTI in the present study was in agreement with other seroprevalence studies carried out in various parts of India. NAT yield in our study was 1 in 628 donations which was comparable to a previous study performed at the same institution [18]. There was high proportion of ELISA positive/NAT negative for HIV (50%) and HBV (15%) in our study (Table 2). This could be either due to low viral load below detection limits of the NAT assay or due to false-positive results in ELISA. The differences in the proportion of ELISA positive/NAT negative for HIV (50%) and HBV (15%) in our study can be explained by the fact that we performed fourth generation ELISA testing for HIV which has a high potential risk for nonspecific reactivity [19].

Transfusion safety begins with healthy donors. A fundamental part of preventing TTI is to notify and counsel reactive donors. Donor notification and counseling protect the health of the donor, prevent secondary transmission of infectious diseases to sexual partners, reduces risk of vertical transmission and provide feedback about the effectiveness of donor selection procedures such as predonation education and medical history [20]. We attempted to contact all 2838 (2.51%) reactive donors about their TTI status either telephonically or by letter. Only 662 (23.3%) reactive donors responded to the notification. In an Indian study by Patel et al. 236 (60.36%) donors showed a positive response following donor notification [6]. In another study by Agarwal et al. involving 416 reactive donors, only 249 (59.8%) donors turned to transfusion facility and attended counseling after receipt of their reactive status [21]. The counseling success rate at large blood center in southern India was 41.18%, 11.11%, and 14.63% for HBV, HIV, and HCV, respectively [22]. Donor response rate in our study was low as compared to other studies from the country. This may be due to poor health care knowledge, social stigma associated with TTIs (especially HIV), and inadequate understanding of implications of screening tests among the general population [21, 23]. Also, as our center is a large volume referral center in India, most of the donors belonged to far-off places; thus distance could be a reason for the donors not reporting back to transfusion facility. Given the large volume of daily donations coupled with limited resources (only one counselor), it is difficult to ensure that every donor had understood the meaning and intent of counseling to the best of his/her intelligence. Another explanation which we think of for low response rate was nonreceipt of postal letters as donors tend to write inadequate postal address in donor registration forms. The study by Kleinman et al. reports that following notification 27% of donors contacted the blood center for further information [24].

One more finding of this study that should be a serious concern for blood transfusion authorities is that only 373 (56.3%) of 662 responsive donors responded to the first call and followed up their attendance at the ICTC or with the physicians they were asked to meet. Rest of the 289 donors (43.7%) was lost to follow up at this very first stage. This raises questions about the way donors are counseled and made aware of the consequences of not taking proper treatment. It is also alarming that only 176 donors (6.2%) of all the reactive donors either were taking treatment or had completed it 6 months after having received notification of their infectious status. We could not find any studies from India to compare this data. The donors who did not turn up to transfusion facility (nonresponders) may continue to donate blood at other centers especially those centers which do not use biometric donor identification, hence posing serious threat to safety of blood supply. This threat is amplified by the fact that though ELISA is the recommended and preferred screening technique, many blood centers still do not have this facility and rely on “rapid kits” which may have high false-negative rate. Donors who are ELISA/NAT reactive elsewhere may escape TTI screening [25].

As per objective 4.16 of the Indian action plan for blood safety, the blood donors are counseled about TTIs prior to donation and are offered the option of knowing their seroreactive status provided they give their consent. Low donor response rate suggest that we are not able to meet this goal with reasonable satisfaction. Another important problem which we encounter in our daily transfusion practice is about donors who are notified because of NAT reactive/ELISA nonreactive status. These donors have their tests repeated by their personal physicians and return with discrepant results as most of the laboratories again screen samples by ELISA. These donors usually demonstrate an angry behaviour in blood bank and question the accuracy of screening performed in blood bank. More often than not despite our best attempts we are not able to explain the meaning of sensitivity and specificity of testing methods to the donors up to their level of understanding. We need to follow up such cases over 6 months as 95% of infected persons will seroconvert in this timeframe [26].

Our study has two limitations. First, we did not perform confirmatory testing of TTIs prior to notification. Second, repeat NAT was not done on the returning reactive donors (although a quantitative viral load is being performed at the referral center, unfortunately the results of which are not available to us). Our study was a small endeavor in determining donor behavior when informed about their reactive status according to results based on screening assays including NAT. Transfusion safety rests heavily on the health of blood donors. To improve donor response rate, we have switched to exclusive telephonic notification to all donors who test reactive in screening tests. Donors should undergo optimal predonation counseling so as to educate them about the risk of infections and the window period. It is the collective duty of transfusion community to inform these donors and do as much as possible to allay their anxiety about reactive result and to advise them about available treatment options. There is an urgent need to formulate the nationally acceptable guidelines for notification of all reactive donors. We have tried to formulate such schema (Figure 1). We expect that other blood centers will carry out such studies and further refine the algorithm.

## Figures and Tables

**Table 1:** Demographic details of donations.

Donor demographics (*n* = 113,014)		
	Number	%
Gender		
Male	108,042	95.6
Female	4,972	4.4
Age group		
18–25	36,390	32.2
26–40	61,367	54.3
41–65	15,257	13.5
Residence		
Urban	78,206	69.2
Rural	34,808	30.8
Donation type		
Voluntary	16,516	14.6
Replacement	96,498	85.4
Donor repeatability		
First time donors	109,669	97
Repeat donor	3,345	3

**Table 2:** Prevalence of TTI markers and comparison of ELISA versus NAT.

Marker	Reactive donors	ELISA+/NAT (%)	ELISA+/NAT+ (%)	ELISA−/NAT+ (%)	Prevalence (%)
HBV	1557	232 (0.21)	1218 (1.08)	107 (0.09)	1.38
HCV	612	269 (0.24)	272 (0.24)	71 (0.06)	0.54
HIV	311	156 (0.14)	153 (0.13)	2 (0.002)	0.27
Syphilis (RPR)	358	NA	NA	NA	0.32
Total	**2838**	**657**	**1643**	**180**	**2.51**

^*^NA: not applicable.

**Table 3:** Donor response rate.

Marker	Reactive donors	Voluntary donors			Replacement donors		
		Notified	Response	%	Notified	Response	%
HBV	1557	143	69	48.2	1414	244	17.3
HCV	612	75	42	56	537	140	26.1
HIV	311	39	5	12.8	272	91	33.5
Syphilis	358	18	4	22.2	340	67	19.7
Total	**2838**	**275**	**120**	**43.6**	**2563**	**542**	**21.2**
